# Understanding parental perspectives on outcomes following paediatric encephalitis: A qualitative study

**DOI:** 10.1371/journal.pone.0220042

**Published:** 2019-09-16

**Authors:** Jennifer Lemon, Jessie Cooper, Sylviane Defres, Ava Easton, Manish Sadarangani, Michael J. Griffiths, Andrew J. Pollard, Tom Solomon, Rachel Kneen

**Affiliations:** 1 Institute of Infection and Global Health, University of Liverpool, Liverpool, United Kingdom; 2 Alder Hey Children’s NHS Foundation Trust, Liverpool, United Kingdom; 3 School of Health Sciences, Division of Health Services Research and Management, City University of London, London, United Kingdom; 4 Tropical Infectious Diseases Unit, Royal Liverpool University and Broadgreen NHS Trust, Liverpool, United Kingdom; 5 Encephalitis Society, Malton, United Kingdom; 6 Vaccine Evaluation Center, BC Children’s Hospital Research Institute, Vancouver, Canada; 7 Division of Infectious Diseases, Department of Pediatrics, University of British Columbia, Vancouver, Canada; 8 NIHR Health Protection Research Unit in Emerging and Zoonotic Infections, University of Liverpool, Liverpool, United Kingdom; 9 Oxford Vaccine Group, Department of Paediatrics, University of Oxford, Oxford, United Kingdom; 10 NIHR Oxford Biomedical Research Centre, Oxford, United Kingdom; 11 Walton Centre NHS Foundation Trust, Liverpool, United Kingdom; University of Birmingham, UNITED KINGDOM

## Abstract

**Background:**

Encephalitis, characterised as inflammation of the brain tissue, is an important cause of acquired brain injury in children. Objective clinical outcomes vary significantly between affected patients, however they do not always correlate with quality of life as reported by parents. The aim of this study was to explore how parents experience and interpret outcomes in relation to their child who has been affected by encephalitis.

**Methods:**

Data were derived from in-depth, semi-structured interviews, with 15 parents of 12 children and young people affected by encephalitis. Paediatric cases were identified from the retrospective arm of the research programme ‘ENCEPH-UK-Understanding and Improving the Outcome of Encephalitis’, and from the prospective UK childhood meningitis and encephalitis cohort study (UK-ChiMES, 2012 to 2016). Data were analysed thematically.

**Results:**

Parents’ perspectives on important outcomes for their child and family changed during the different stages of the encephalitis illness trajectory: from acute illness, recovery and rehabilitation, then reintegration into everyday life. Parents’ understanding of their children’s overall outcome was informed by their own experiences, involving comparisons with other children and reflections on their child’s problems before, during and after the acute illness.

**Conclusion:**

Outcomes in paediatric encephalitis need to be understood in terms of the context of the patient and family experience as well as the timeframe of recovery. The research highlights the need to include more patient, parent and/or carer reported outcome measures during patient assessment, and that assessment should be repeated during recovery as family concerns change. In the longer term, these parameters could be included in clinical and rehabilitation practice to further support child recovery.

## Introduction

Encephalitis is a serious neurological condition, which can result in death or permanent disability [1]. The incidence in children is 10.5–13.8 per 100,000 which equates to approximately 1–2 paediatric cases expected at an average district general hospital in the United Kingdom per year [1, 2]. The condition is characterised by brain tissue inflammation, resulting from a range of infections and immune-mediated processes [3]. At illness onset, there is usually severe deterioration in the physical and mental functioning of those affected, with between 40–52% of paediatric patients requiring intensive care unit (ICU) admission [4–6]. Symptoms include: altered level of consciousness or abnormal behaviour, fever, vomiting, seizures, communication difficulties, limb weakness and headache [1, 4].

Although mortality from paediatric encephalitis has decreased over past decades to <4% in high resource settings, recovery is highly variable [6, 7]. Follow-up studies have reported persisting sequelae in 42–63% of children, which include seizures, motor weakness, developmental delay, reduced intelligence quotient (IQ), vision and hearing problems [4, 8, 9]. Cognitive and behavioural problems are commonly reported but can be subtle and may not become apparent until after the acute illness [4, 5]. Furthermore, the condition can recur in children with autoimmune aetiologies, including acute disseminated encephalomyelitis (ADEM) and antibody mediated encephalitis [10, 11]. Taken together, this means there is a high degree of variability and uncertainty surrounding recovery and the clinical outcomes for children following encephalitis.

Porter [12] stated that “achieving good patient health outcomes is [understood as] the fundamental purpose of health care”. However, it is acknowledged that the definition of what constitutes a ‘good’ clinical outcome has been poorly outlined in the medical literature [12]. Furthermore, insufficient attention has, historically, been dedicated to outcome selection in the context of clinical trial design, with patient’s perspectives being rarely incorporated [13]. In relation to paediatric encephalitis, follow-up studies lack consistency with regard to types of outcomes measured [14], and clinical trials have predominantly reported on outcomes relating to acute disease activity, physical impairments, or functional ability [15–17].

In some encephalitis trials, validated questionnaire-based tools, such as the Liverpool Outcome Score (LOS) [18] and the paediatric Glasgow Outcome Score (GOS), have been used to assign an outcome severity category to participants [15, 16]. Assigning categories in these ways enables ‘objective’ comparisons of clinical outcomes between patients, however, it may not reflect the complexity of outcomes for children and their families within the context of their everyday lives. For example, a recent study found that less than half of encephalitis patients who made a ‘good’ functional recovery on the GOS reported Health Related Quality of Life (HRQoL) scores comparable to the general population [19]. Despite the inference that patients and carers may make sense of their overall outcome in different ways, there are few detailed studies which investigate how outcomes in paediatric encephalitis, or other acute brain injuries, are understood and experienced from the perspectives of patients and their families. The aim of this study was to understand, from the parents’ viewpoint, the impact of encephalitis on their child and family life, which outcomes they felt were most important and how their perspective on these issues changed over time.

## Methods

### Background to the study

This paper reports on qualitative interview data from the Core Outcomes Study. To achieve the study aim, a qualitative methodology, involving in-depth semi-structured interviews was chosen. The use of qualitative methodology within research into health experiences enables detailed understanding of what is important to the participant, including the meanings they attach to health and illness [20]. Qualitative approaches have been successfully employed in other studies which sought to identify which health outcomes are important to parents of children with chronic health conditions and disabilities [21, 22]

The Core Outcomes Study was a sub- study within the UK Childhood Meningitis and Encephalitis cohort study (UK-ChiMES) and ENCEPH-UK, a National Institute for Health Research (NIHR) programme grant for applied research on ‘Understanding and Improving the Outcome of Encephalitis’. Further details are given below and full information can be accessed from the ENCEPH-UK website www.encephuk.org. This study was granted ethical approval by the National Research Ethics Service (NRES), East Midlands Committee (11/EM/0442), protocol number UOL000755.

### Participants

Participants in this study were parents of children and young people with encephalitis who were identified and recruited from two inter-related cohorts; Firstly, from the retrospective ENCEPH-UK cohort, in which cases of proven encephalitis at any time between 2005 and 2012 had been retrospectively identified and recruited. Secondly, from UK-ChiMES (the prospective paediatric arm of the ENCEPH-UK Programme along with a paediatric meningitis cohort study led by the University of Oxford), which recruited suspected encephalitis and meningitis cases between December 2012 and March 2016, from 31 hospital sites across the UK. Case definitions for encephalitis were the same across both cohorts (S1 Appendix). For this Core Outcome Study, only parents of children and young people (<16 years at diagnosis), who met the definition for clinical encephalitis, with or without a specific aetiology, were considered for inclusion.

Cases of encephalitis were purposively sampled from the retrospective and prospective cohorts to ensure a diverse and representative sample in terms of the following characteristics: a) spread of ages at onset of acute encephalitis (median: 3 years, 7 months; range: 0 days to 15 years, 10 months); b) varying encephalitis aetiologies; and c) varying duration of time since onset of acute encephalitis (median time between acute illness and parent interview: 2 years, 1 month; range 9 months to 5 years, 6 months). Within this study, 11 interviews were conducted with 14 participants. One further parent was interviewed for the End User Experience study [23], another sub study of ENCEPH-UK, and consented for her transcript to also be analysed as part of this Core Outcomes Study. This resulted in a total of 12 interview transcripts, from 15 participants.

### Data collection

Potential participants were initially contacted by phone or email, and written information was sent out to those who were interested in participating. Data was collected by means of semi-structured interviews, between December 2013 and February 2014. Semi-structured interviews were chosen, as they enable in-depth exploration of specific topics important to the research question, while also ensuring flexibility to allow for discussion of unanticipated issues initiated by participants [24]. A topic guide (S2 Appendix) was used, which included prompts relating to: parents’ experiences of their child being diagnosed and treated for encephalitis, the impact of encephalitis on their child and family, and parents’ perspectives on their child’s future. Prior to commencing data collection, the topic guide was reviewed and revised, following feedback from parent members of *The Encephalitis Society*, and was tested in a pilot interview.

All interviews were conducted in the participants homes, at their request. If both parents were interested in participating, they were given the option to have joint or individual interviews- all preferred to be interviewed together. No one other than the interviewer and the participant/s were present during the interview. The interviewer was JL, a female medical doctor, who had received training in qualitative methodology. To maintain the emphasis on investigating parents’ perspectives on outcomes and to avoid undue focus on medical aspects of the cases, JL introduced herself as ‘a researcher from the University of Liverpool’. Her medical status was openly disclosed if this came up in part of conversation at the end of the interview. All participants provided written, informed consent at the time of the interview. The flexible style of the interviews allowed for participants to decide how much information they wanted to divulge about sensitive topics. Interviews lasted between 40 minutes and two hours fifteen minutes. The data was limited to a sample size of 12 interviews due to the in-depth nature of parents’ narratives, which is in keeping with the fact that qualitative research aims for depth rather than breadth of data.

The interviews were audio recorded and transcribed verbatim by JL and an experienced typist, who had signed a confidentiality declaration. Identifiable details, including all names and locations, were anonymised during transcription. Participants were given the option to have a copy of their anonymised transcript returned to them for checking.

### Data analysis

Transcripts were transferred into NVivo 10, a software programme for organising and analysing qualitative data. Thematic analysis was used to identify and analyse patterns across accounts, in relation to parents’ understanding and experience of their child’s outcomes. Thematic analysis can help generate unexpected insights from the data and allows the researcher to gain an understanding of commonalities as well as differences within the data set [25]. During the analysis, particular attention was paid to the ways in which parents understood their children’s outcomes over time, and how they constructed their perceptions of what constituted a ‘good’ or ‘bad’ outcome for their child.

The transcripts were subject to initial coding, by one researcher (JL), which involved creating codes in the form of key descriptive phrases for patterns within the data. These codes were then used to produce a coding framework in which codes were grouped together under initial themed titles. The final themes were developed by an iterative process of moving back and forth between the data and the codes and developing overarching concepts and explanations for patterns and accounts within the data [25]. Themes were discussed, revisited and refined with another qualitative researcher (JC), a medical sociologist who has extensive experience of qualitative methodology, to describe and explain the content and meaning of each account.

In the results, below, data is presented in the form of three cases. The use of cases to present data on encephalitis patients has been successfully used in other publications [23, 26, 27]. Presenting the data as cases enables illustration of how parental priorities change over time, in a way that using individual quotes from multiple participants would not allow.

## Results

The 15 participants (11 mothers, three fathers and one step-father) were parents of 12 children and young people (cases), who had been affected by encephalitis. Encephalitis aetiologies included autoimmune encephalitis (n = 4), viral encephalitis (n = 3), clinical encephalitis, unknown aetiology (n = 2), bacterial encephalitis (n = 1), acute necrotising encephalitis of childhood (n = 1), and ADEM (n = 1). Three of the cases had made an apparent full recovery from their encephalitic illness as reported by their parents. The remainder had a range of ongoing difficulties. Demographic characteristics of the cases and their parents, and a summary of reported problems from the parent’s perspective, are shown in Table 1.

**Table 1:** Summary of characteristics for parent participants (n = 15) and their children (n = 12).

Parent (participant) characteristics			Child/ young person (case) characteristics						
No.	Relationship to child	Age	ChiMES/ ENCEPH-UK Study number	Encephalitis aetiology classification	Gender	Age at illness onset (years)	Age at interview (years)	Time since encephalitis at interview (months)	Persisting clinical problems at time of interview as reported by parents
***Retrospective cohort***									
1*	Mother	33	007	Viral encephalitis (parainfluenza)	F	4.3	9.8	66	Wheelchair dependent, incontinent, problems manipulating objects
2*	Mother	37	080	Autoimmune encephalitis (anti-NMDA antibodies)	F	2.8	5.2	29	Difficulty sleeping at night, slightly slower to do school work than previously
3*	Mother	46	081	Viral encephalitis (HSV)	M	8.8	13.4	53	Difficulty with fine hand movements and coordination, short term memory loss, concentration difficulties, headaches, fatigues easily, heightened emotions
4	Mother	46	079	Autoimmune encephalitis (anti-VGKC antibodies)	F	13.6	16.3	33	Apparent full recovery
5	Mother	52	084	Autoimmune encephalitis (anti-VGKC antibodies)	F	14.8	16.3	17	Behavioural change
6	Father	53							
7	Mother	41	024	Bacterial encephalitis (Group B Streptococcus)	M	0.2	6.6	77	Wheelchair dependent, incontinent, not able to use upper limbs, no speech, seizures, visual impairment, needs full help with self-care needs
8	Mother	39	082	Clinical encephalitis (aetiology unknown)	M	1.9	3.8	21	Left sided weakness, heightened emotions
9#	Mother	38	013	Viral encephalitis (HSV)	M	0.0	5.8	68	Autistic traits, sensory behaviours, behavioural problems, clumsy
***Prospective cohort***									
10	Mother	35	168	ADEM	M	1.2	1.7	7	Apparent full recovery
11	Father	37							
12	Father	28	001	Autoimmune encephalitis (anti-NMDA antibodies)	M	2.3	3.3	11	Apparent full recovery
13	Mother	53	110	Clinical encephalitis (aetiology unknown)	F	15.8	16.6	9	Headaches, fatigues easily, motion sickness
14	Step-father	52							
15	Mother	39	040	Clinical encephalitis (ANEC)	F	5.2	6.2	11	Visual impairment, memory difficulties, left sided weakness, fatigues easily, heightened emotions

ANEC-Acute necrotising encephalitis of childhood, ADEM-Acute disseminated encephalomyelitis, HSV: Herpes simplex virus, NMDA: N-Methyl-D-aspartate, VGKC: Voltage gated potassium channel,

*Highlights the three participants whose accounts are written up in detail in the results,

^#^ indicates the parent who was interviewed for the ENCHEPH-UK programme ‘End User study’ and consented for her transcript to be analysed for this study

The three cases we present below include children of different gender, ages and encephalitis aetiologies. We also wanted to ensure diversity with regard to type and severity of problems experienced post-encephalitis: case 1 has severe physical impairments; case 2 has made an almost full physical recovery but has ongoing cognitive problems that impact on his daily functioning; case 3 has a few minor resulting problems, however in the parents own words she felt that the encephalitis now had ‘no impact’ on her daughter. Case 3 was therefore selected to represent those children who had made a good/full recovery following encephalitis. As well as representing a range of characteristics, these particular cases were selected as, when taken together, their narratives depict, most typically, the accounts of all the participants in this study and serve to evidence the themes identified.

### Case 1-Hannah and Cynthia

Hannah was four years old when she developed encephalitis, associated with a parainfluenza infection. Her story is told by her mother, Cynthia, who was interviewed five and a half years after Hannah’s acute illness, when Hannah was living with her mother, father and younger brother. Although the encephalitis did not affect Hannah’s learning ability or personality, she is a full-time wheelchair user, has reduced upper limb strength and is incontinent.

Cynthia described how she initially took Hannah to hospital after she became concerned that her ‘arms and legs were cold’ and she was ‘throwing herself around’. At her local hospital, Hannah was intubated to go for a computer tomography (CT) scan and was subsequently transferred to an intensive care unit (ICU) at another hospital. When Cynthia discussed her memories of Hannah’s time in ICU she recalled: ‘all these lines going into her body’ and the ‘machines around her’. At this time, Cynthia was concerned that Hannah’s kidneys were not functioning properly, and she had an overwhelming fear that she ‘would lose’ her daughter. She was in disbelief at the situation, since the illness had come entirely ‘out of the blue’: Hannah had been in good health a few days earlier.

After several weeks, Hannah’s condition improved and she was moved to the high dependency unit (HDU). Cynthia recalled a moment, on HDU, when Hannah moved her head and described her hope at seeing this return of the most basic function in her daughter: ‘she wasn’t moving any other part of the body, but [at least] she was moving’. Cynthia explained how she then started questioning the extent to which Hannah would recover from encephalitis. Her main concern at the time was whether her daughter would be able to speak and eat again. During her time on HDU, Cynthia remembered a period where Hannah became ‘really upset’ and attributes this to her being ‘aware but not understanding’ what was happening to her. After 2 months, Hannah was transferred to an inpatient rehabilitation unit in her local hospital. Cynthia recalls that her priorities at this time were for Hannah to improve her clarity of speech, sitting balance and strength and to get her back home.

After discharge Hannah had a slow transition back to mainstream school and took a long time to settle into the classroom. Cynthia reflected how, although things have changed over time, there is ‘always a different concern’ to be dealing with. For example, she discussed Hannah’s ongoing problems in terms of the impact on daily life: her weak hand grip means she has difficulty with handwriting and manipulating small objects, wearing incontinence pads results in Hannah often missing playtime at school because she is being changed by her teachers. Although Cynthia reported that having an electric wheelchair has increased Hannah’s independence, she recalled scenarios where Hannah had difficulty accessing buildings or activities at school and in the community, including having swimming lessons in a different pool to her friends.

Cynthia explained how Hannah’s illness has had a huge impact on family life and she has given up work in order to care for her daughter. She also described the difficulties of trying to balance attention between Hannah and her younger brother and expressed concern that there are some things he misses out on. Regarding the future, Cynthia reported that she was concerned about finding the right secondary school for Hannah and hoped she would be accepted by her peers and be content and comfortable in life. Cynthia summarised their situation by saying that: ‘we have to be grateful…for her being here, being able to talk, able to eat, just little things, able to breathe on her own and just being happy’.

### Case 2-Freya and Sarah

Freya was two years old when she was diagnosed with encephalitis caused by anti-N-Methyl-D-aspartate (NMDA) receptor antibodies. Her mother, Sarah told her story three years later, when Freya had made an almost complete recovery after a long initial hospitalisation. At this time, Freya was living with her parents and teenage sister.

The first sign that Freya was suffering from encephalitis was when she had a seizure and was airlifted to hospital. Sarah recounted how, the following day, Freya started to ‘dribble’ and ‘her leg stopped working’. From then on, according to Sarah, Freya changed ‘from being a normal two-and-a-half-year-old, to not being able to speak, walk or even sit up’. Sarah reported that it was about four weeks before the hospital found out the cause of Freya’s encephalitis. During this time, Freya’s body was affected by ‘spasms’, causing her hands and feet to ‘twist’ into awkward positions. She was unable to communicate and did not recognise her parents, even physically attacking them. Sarah said it was like a ‘devil had got inside of her body’ and her biggest fear during this time was that Freya would die.

During her time in hospital, Freya’s awareness slowly improved. As she became more aware of her surroundings, Sarah reflected that this made the long plasmapheresis (blood cleaning) treatment sessions increasingly more unbearable for the family: Freya’s uncontrollable movements meant her parents had to hold her down during these sessions, to make sure she didn’t ‘rip out’ her intra-venous lines. Several weeks later, Freya started to smile, which signalled to Sarah that her ‘little girl [was] coming back’. At discharge, Freya was unable to walk or talk properly, but Sarah was hugely relieved that she remembered their family house. Her recovery from thereon was a very quick acquisition of skills, which Sarah helped her to relearn.

Although Freya made a rapid physical recovery, Sarah explained how the encephalitis medication had suppressed Freya’s immune system. This resulted in numerous trips back to hospital every time she had a temperature and a delayed start to nursery, as Sarah was concerned about her catching an infection.

Sarah described how, at the time of the interview, five-year-old Freya loved school and playing with friends. She explained how Freya had ongoing problems with poor sleep and was taking a slightly longer time to learn at school when compared with her classmates. However, despite these problems, Sarah felt the encephalitis had ‘no [ongoing] impact’ on her daughter, going on to explain how she felt lucky at Freya’s outcome, since, as she put it: ‘I didn’t even think she would live at one point, or go to school’. When considering the future, Sarah reflected: ‘I just want her to have a happy life … I would love to think that it [the encephalitis] will never come back again, but I don’t really know that’.

### Case 3-Michael and Kathy

Michael was eight years old when he developed Herpes simplex virus encephalitis. His mother Kathy, who works in management, was interviewed five years after her son’s hospitalisation. At this time, 13-year-old Michael was in secondary school and living with his parents and brothers. Although he had made a good physical recovery, Kathy described how poor memory and planning skills post-encephalitis illness had an ongoing impact on Michael’s day to day home and school life.

Kathy described how Michael first showed signs of illness when he started getting headaches and subsequently became drowsy, with ‘slurred speech, like he was drunk’. Kathy took Michael to several health professionals before he was admitted to hospital and treated for encephalitis. Whilst he was, in Kathy’s words, ‘semi-unconscious’ in the emergency department, she recalls thinking: ‘I don’t care what he’s like, just don’t let him die’. The next morning, following emergency treatment and stabilisation, Michael was more awake, however Kathy likened him to a new born baby: ‘all floppy and slobbering from his mouth’. At that point, she was reassured that he wasn’t going to die but prayed ‘please God don’t let him be this bad for ever’.

On the ward, Michael made slow improvements, but was distressed by frequent severe headaches. Kathy remembers Michael’s frustration when he struggled to recall words and feed himself and recounted his distress when his brothers made fun of his slurred speech. After three weeks, Michael was discharged home. At this point he was mobile but had frequent falls, fatigued easily and needed help with personal care. As the months passed, these problems slowly improved and he underwent a phased return to his primary school.

Although Michael has experienced difficulties with memory and organisational skills since diagnosis, according to Kathy, the impact of these problems on his learning and wellbeing were amplified when he left the supportive ‘cocoon’ of primary school. Kathy explained that at secondary school, Michael has struggled academically, and describes himself as ‘stupid’. Furthermore, he regularly gets in trouble for messy handwriting, being late and forgetting his homework. Kathy attempted, on multiple occasions, to educate school staff about Michael’s condition, but felt that they only took notice when she threatened to remove him from the school. In her opinion, the lack of any obvious visible signs of Michael’s problems reduces people’s understanding of his condition.

Kathy feels that she has become ‘over-protective’ of Michael since his illness and described how the family have altered their routines to support him, including helping him get washed and dressed in the mornings and arranging for him to get a lift or taxi home from school instead of the bus. Although Michael’s older brothers sometimes comment that Michael receives more attention than they do, they also protect and look out for him at school. Kathy expressed that they are fortunate, to have good ongoing support from their extended family.

When asked for her opinion on Michael’s overall outcome following encephalitis, Kathy admitted that she used to grieve for the ‘loss of the child she had’, explaining that comparing her son to other 13 year olds highlights his difficulties and reduced independence. However, despite his problems, Kathy described her son’s recovery as a ‘miracle’, which she explained in contrast to another child who had encephalitis on Michael’s ward, who was left ‘paralysed and incontinent’.

### Interpretation of the findings: Understanding the significance of time and context in encephalitis outcomes

The cases presented above depict the typical experiences of parents regarding their child’s outcomes following encephalitis. In the sections which follow, we interpret the findings within themes by, firstly, describing how parents’ perspectives on their child’s outcomes shift in relation to their experiences at different temporal stages of the illness trajectory, namely those we classify as the: acute, recovery and reintegration stages. We then examine the ways in which parents use points of comparison, specifically between their own and other children, and problems faced at different times in the illness trajectory, to contextualise their understanding of their child’s overall outcome following encephalitis.

#### Acute illness- focusing on the dysfunctional body

When their previously healthy child was hospitalised with encephalitis, parents focused on the chaos that encephalitis had wrought on their child’s body and mind, disrupting their child’s ability to function normally. They recounted the loss of their child’s most basic physical and mental functions, including: reduced awareness of their surroundings, the inability to move body parts, communicate, see or even breathe on their own. For some parents, including Sarah, this loss of function was coupled with an understanding that their child’s body and mind had become chaotic, evidenced by the presence of fits, strange hand or leg movements and change in behaviour. Moreover, particularly for those parents, like Cynthia, whose children were treated in ICUs or HDUs, they saw their childrens dysfunctional bodies adorned with medical devices. These technologies, consisting of lines, tubes and machines, which were sustaining their child’s bodily functions, further emphasised the severity of their child’s condition. Almost all parents described how, at this stage of their child’s illness, they had an overwhelming fear that their child would die. However, as children started to recover from the acute phase of encephalitis, parents emphasised how new priorities developed.

#### Recovery and rehabilitation-focusing on outcomes beyond survival

Many parents described a moment during their child’s hospitalisation, when they realised their child was showing signs of recovery. This turning point, as one parent labelled it, corresponded with the return of some element of their child’s physical or mental functioning, such as breathing for themselves, regaining sight, moving limbs, smiling and speaking. We see this reflected in the cases, with Hannah’s first movements and Freya’s first smile. Several parents, including Kathy, described how, only after they were reassured that their child was not going to die, could they start to consider outcomes beyond their child’s immediate survival.

However, while parents recounted their relief when they realised that their child was starting to improve, some reflected on the fact that, as their child became more aware of their surroundings, their suffering conversely increased, as described in all the cases. These concerns were characterised in two ways. Firstly, parents including Kathy, highlighted the distress their child would experience as a direct result of the effects of encephalitis, such as suffering from headaches and the inability to control their own body. Secondly, they described how children were troubled by increasing awareness of painful medical interventions, such as cannulation. Many parents in the study found it extremely upsetting to witness the pain and suffering of their (increasingly aware) child. However, the realisation that their child was improving enabled parents to focus on their child regaining basic independent physical and mental functions, like walking, talking and eating and start to consider their reintegration back into their everyday life.

#### Challenges of reintegrating into everyday life after encephalitis

Following discharge, parents described how, over time, they and their child resumed everyday activities including returning to employment and school. Below, we characterise the key challenges, as highlighted by parents, during this period and extending to the time of interview, in relation to the reintegration of children and families into the routine of daily life following encephalitis.

Sequelae within a social context: At this stage of the illness trajectory parents discussed their children’s problems post-encephalitis within the context of their everyday lives, in four main ways. Firstly, parents described their child’s difficulties in terms of how they affected independence with regard to moving around and self-care activities such as dressing, toileting and feeding. Maximising independence was identified as a particularly important priority for older children and their parents, as highlighted by Cynthia and Kathy. Secondly, academic progress at school or nursery was a key concern, with some parents discussing the detrimental impact of encephalitis sequelae on learning, as highlighted in Michael’s case. Good school achievement acted to reassure other parents that the learning ability of their child had not suffered. Thirdly, parents including Cynthia and Kathy, described the impact of encephalitis sequelae on their child’s activity participation within the school and community environment. For example, parents focused on the extent to which their child’s illness had an impact on their ability to join in activities with their peers and make and maintain friendships. Finally, parents discussed their child’s problems in relation to the impact on their overall wellbeing and happiness. As expressed by Sarah and Cynthia, seeing their children happy and content, regardless of their specific difficulties, was stated by many parents as their most important priority.

Mediating factors and parental advocacy: *M*odifiable external factors, including availability of resources, adaptations, support and the attitudes of others were felt by many parents to mediate the broader social impact of their child’s encephalitis sequelae. For example, lack of wheelchair facilities hindered Hannah’s participation in school swimming lessons, and limited understanding from teachers of Michael’s memory and organisation difficulties were felt by his mother to negatively impact on his learning and happiness. Many parents, particularly of children with less visible disabilities, described how additional resources (e.g. equipment for the home or school, extra teaching support) for their children were not always forthcoming. Moreover, when parents, like Kathy, saw their children suffering due to lack of resources, or un-supportive individuals/ organisational environments, they often adopted an advocacy role, taking it upon themselves to fight for extra support where needed. They did this by communicating with health/social care/educational services, to educate professionals about the impact of encephalitis for their child and secure extra resources, equipment or support. Parents, including Kathy, often described how this advocacy role was ongoing, as their child’s needs changed over time and they started new activities or moved to new school environments.

Family impact: Parents also discussed the various ways in which encephalitis had impacted on themselves and other family members. In the initial phases of the illness, parents frequently described how they put their own day-to-day lives on hold whilst they focused their attention on their critically unwell child. Once their child started to recover, it was often necessary to resume employment and other activities, however the impact of their child’s condition continued to affect parents’ work and family lives. A concern for many parents, including Cynthia, was balancing the attention they gave to their affected child and their ‘healthy’ siblings, as well as being able to juggle the usual routines of work and family life. As a result, some parents needed to alter their own daily routine and work schedules to accommodate their child’s additional needs and the advocacy activities described above. Some parents, such as Cynthia, gave up work completely, to care full-time for their newly disabled child.

Parents’ understanding of their child’s outcomes once they have returned to their everyday lives are therefore narrated in relation to, a) their child’s ability to function within their wider social world, b) the resources and support which mediate this functioning, and c) the extent to which these problems impact on parents and other family members.

#### Contextualising current outcomes

In addition to considering outcomes with reference to the day-to-day impact of problems described above, parents also assessed their child’s current situation by making comparisons to other children, and reflecting on their child pre- and post-encephalitis. 1) Reference to other children: comparisons were made to peers and siblings to understand how their child differed in relation to ‘normal’ development. Where children had ongoing problems, these comparisons often acted to highlight the difficulties their own children experienced, as illustrated by Kathy. Additionally, parents often compared their child to other encephalitis cases they had read about, or children they had met in hospital as a way of judging their child’s outcome, as we saw with Kathy’s comparison of Michael to another child affected by encephalitis. 2) Reflecting back in time: Parent’s would reflect, sometimes negatively, on the differences between their ‘healthy’ child, pre-encephalitis, and how their child was in the present. Kathy and the two parents whose children were babies at the time of encephalitis also referred to loss of the child that they might have had in the absence of encephalitis. In contrast, when parents reflected upon their child’s acute illness and hospitalisation, they made more positive evaluations of their child’s present outcome, with many, including Cynthia and Sarah, commenting on how lucky they felt that their children survived 3) Orientations to the future: Parents highlighted a range of concerns about how encephalitis might affect their child in the future. The uncertainty surrounding future outcomes impacted parents evaluation of their child’s current situation in three specific ways: a) Some parents expressed general worries surrounding their child’s future independence, employment and happiness, as voiced by Cynthia; b) many parents, particularly where their children had autoimmune encephalitis, were only cautiously optimistic about the future, as they worried about recurrence of illness, a concern that Sarah highlights; c) Some parents of young children, even those with an apparent full recovery, made reference to uncertainty about how their child would develop, concerned with whether problems would worsen or new ones become apparent over time as they became older.

Parents evaluations of their child’s outcome therefore involved making comparisons to other children and an understanding of their current situation in relation to what could have happened or what still might happen for their child.

## Discussion

In this study, parents discussed the varying ways in which encephalitis impacted on their child and family. The findings show how parents’ concerns about their child’s outcome evolved over the course of the illness trajectory. Furthermore, we have highlighted how parental perspectives on outcomes, following acute brain injury secondary to encephalitis, are informed by comparing their child’s actual outcome in relation to theoretical scenarios. Many of the specific contextualised concerns described by our parents have been previously highlighted in other encephalitis narratives [27] and broader qualitative literature on acquired brain injury in children (which predominantly includes traumatic brain injury) [28, 29]. We have developed the existing knowledge base further by showing how these concerns specifically relate to parental perception of outcomes for their children.

Crucially, the findings have highlighted how parental perception on outcomes are underpinned by the particular temporal trajectory of a condition like encephalitis. As the child moves through different stages of the disease (acute, recovery, reintegration into the social world), the parental focus on outcomes shifts in parallel; their initial ‘biomedical’ focus on survival and the impaired body is replaced over time by attention to a child’s independence and ability to participate in everyday social routines. It is important to note that several ‘outcome frameworks’ have been outlined in wider literatures, which use hierarchies to model the complex interplay between different ‘levels’ of outcomes: differentiating between biomedical, clinically measured outcomes and those which relate to broader social concepts such as independence, participation and well-being [21, 30–32]. Whilst the findings from this study broadly fit with the notion of outcomes as defined in these frameworks, such as the International Classification of Functioning Disability and Health, we have, importantly, demonstrated that outcomes following encephalitis need additionally to be understood as temporally constructed, where the relative importance of biomedical compared with broader social outcomes have varying importance depending on the stage of the illness trajectory. It is likely that these findings may also apply to children with acquired brain injury from other causes which share a similar illness trajectory, with an acute event/illness and then a gradual recovery with variable ongoing clinical sequelae e.g. stroke and traumatic brain injury.

In the context of encephalitis, understanding how people ‘make sense of the impact of illness within a social context’ [27] is acknowledged to be of key importance. Analysis of narratives from parents and patients affected by encephalitis have previously highlighted how stories are frequently told in terms of life before and after encephalitis, sometimes also reflecting on the loss of a life they might have had [27]. This study builds on the existing encephalitis literature by illustrating how each parent’s unique experiences and perspective acts to shape perceptions of the overall outcome for their child. This means that an objectively ‘good’ or ‘bad’ functional outcome to a clinician or researcher may not be considered in the same way by parents, who reflect on how their child was pre-encephalitis and how they might have been, whilst also living with the day-to-day consequences of a child affected by encephalitis and the fear of what might happen in the future. Our findings thus provide insight into why objective clinical outcomes do not fully correlate with parental reported HRQoL, following childhood encephalitis [19].

### Study limitations

Although modest in size, ours is the only known study to explore parental perspectives on outcomes following childhood encephalitis. Access to a large cohort of paediatric encephalitis cases through the UK-ChiMES and ENCEPH-UK studies, enabled accurate classification of encephalitis aetiologies and allowed purposive sampling to ensure diversity of characteristics within our sample. Although we analysed data from all encephalitis cases together, it was noted that the four children with autoimmune encephalitis and the child with ADEM generally made good recoveries: three of these five cases were felt by parents to have no resulting sequelae, unlike the children with other encephalitis aetiologies. However, these children often had severe and protracted acute illnesses. Furthermore, parents reported additional concerns relating to side effects of medication and fear of future recurrence, providing some suggestion that parental priorities for this group may differ in some ways to the other encephalitis aetiologies. Further research, using larger samples within each aetiology, would help to explore potential differences and similarities further. The finding that parental priorities on outcomes change over time could be further validated by longitudinal, prospective research, to enable serial interviews at different stages of the illness trajectory. Finally, although obtaining parental perspectives is important in its own right, their evaluation of the relative importance of some outcomes may differ to those of their children [21]. Future research in this area should also include the views of children affected by encephalitis.

### Implications for practice

Findings from our study have illustrated the importance of understanding encephalitis outcomes from the perspective of parents. However, as previously discussed, outcomes in previous encephalitis research have predominantly focused on objective clinical measures [8, 15, 16]. Within the wider healthcare and research community, there have been intensified efforts in recent years to capture outcomes from the patient’s perspective, as evidenced by increased use and development of Patient Reported Outcome Measures (PROMs) and Core Outcome Sets (COS). PROMs are tools used to record the patients’ view of their symptoms, functional/ social impact of illness or HRQoL [33], whilst condition-specific COS, developed in consensus with patients/families, document a standardised list of meaningful outcomes to be measured in clinical trials [13]. In relation to encephalitis, more widespread use of PROMs and development of a COS for encephalitis would enable the views of patients and their families to be routinely incorporated into clinical practice, rehabilitation programmes and research studies. This would enable the extended clinical team to target the achievement of more patient/family centred outcome measures during recovery.

In addition to considering which outcomes are important to measure and how to do this, we have shown that it is also important in encephalitis patients to consider the timing of outcome measurement, which has implications for trial design. For example, during hospitalisation, measurement of mortality and clinical/biomedical outcomes would be important to parents, whereas following discharge, measurement of outcomes which are influenced by social factors, such as quality of life, participation in daily activities and family/carer stress would better represent the impact of the condition on the child and family’s life. Furthermore, knowledge of how parental priorities evolve over time is useful for health care professions working with this patient group to better understand which concerns might be important to the family at different stages of the illness. Future longitudinal research looking at long term outcomes in children with encephalitis should incorporate a qualitative component with patients and their families, in order to ensure that any standardised measures of ‘outcomes’ are triangulated and compared with the experiences of patients and their families.

## Supporting information

S1 Appendix Encephalitis case definitions used in the ChiMES and ENCEPH- UK studies. (PDF)

S2 Appendix Core Outcomes Study topic guide. (DOCX)
