# Supplementary material for: Understanding parental perspectives on outcomes following paediatric encephalitis: A qualitative study
Source: PLoS One. 2019 Sep 16;14(9):e0220042. doi: 10.1371/journal.pone.0220042 (PMC6746349; doi:10.1371/journal.pone.0220042)
Supplement: S1 Appendix — (PDF) [file pone.0220042.s001.pdf]

S1 Appendix. Encephalitis case definitions used in the ChiMES and ENCEPH- UK studies

Suspected Encephalitis

Acute or sub-acute (<4 weeks) alteration in consciousness, cognition, personality or behaviour* persisting for more than 24 hours

Plus ANY two of:

a. Fever (≥38ºC) / Prodromal illness – acute or sub-acute

b. Seizures: New onset

c. Focal Neurological Signs – Acute or Sub-acute onset.

- Focal weakness
- Oromotor dysfunction
- Movement disorders** including Parkinsonism***
- Amnesia

d. Pleocytosis: CSF white cell count >4 cells/µl

e. Neuroimaging (CT/MRI) compatible with active encephalitis

f. EEG compatible with active encephalitis

OR

Clinical suspicion of encephalitis of any cause, including non-infective causes

OR

Clinical suspicion of encephalitis but results of investigations unknown

OR

Clinical suspicion of encephalitis or meningitis, but patient died before investigations completed

* personality / behaviour change includes: agitation, psychosis, somnolence , insomnia, catatonia, mood lability, altered sleep pattern and (in children): new onset enuresis, or irritability,
** Movement disorder includes chorea, athetosis, dystonia, hemiballisms, stereotypies, orolingual dyskinesia and tics
***bradykinesia, tremor, rigidity and postural instability

Clinical Encephalitis

Suspected Encephalitis (as defined above)

PLUS

No other unifying diagnosis

PLUS one or more of

Neuroimaging compatible with active encephalitis

OR

EEG compatible with active encephalitis

OR

Patient deceased prior to investigation completion with no other diagnosis

**Bacterial encephalitis**	Clinical encephalitis<br><br>PLUS<br><br>Bacterial pathogen identified from CSF<br><br>OR<br><br>Bacteria identified from blood via culture, antigen detection or PCR attributed to encephalitis
**Viral encephalitis**	Clinical Encephalitis<br><br>PLUS<br><br>Viral pathogen identified from CSF<br><br>OR<br><br>Virus identified from blood via PCR attributed to encephalitis<br><br>OR<br><br>Managed as viral encephalitis
**Acute disseminating encephalomyelitis (ADEM)**	Clinical Encephalitis<br><br>PLUS<br><br>Demyelination seen on neuroimaging consistent with ADEM
**Autoimmune encephalitis**	Clinical Encephalitis<br><br>AND<br><br>Positive auto antibodies consistent with autoimmune encephalitis<br><br>OR<br><br>Imaging consistent with autoimmune encephalitis such as limbic encephalitis<br><br>OR<br><br>Labelled and managed as autoimmune encephalitis
**Labelled as Encephalitis**	Suspected encephalitis<br><br>AND<br><br>Labelled as encephalitis on discharge diagnosis<br><br>AND<br><br>No other attributed aetiology

**Meningitis with Encephalopathy**	Suspected encephalitis<br><br>AND<br><br>Pleocytosis: CSF white cell count >4 cells/µl<br><br>AND<br><br>Not labelled as encephalitis<br><br>AND<br><br>Neuroimaging and EEG do not suggest encephalitis or have not been performed
**Neuroimaging consistent with encephalitis**	Neuroimaging whereby direct inflammation is seen of the brain parenchyma OR vasculitis of the intracerebral vessels OR any subdural pus
**EEG consistent with encephalitis**	Slowing of the background activity or seizure activity

CSF – cerebrospinal fluid; PCR – polymerase chain reaction; EEG – electroencephalogram; CT – computed tomography; MRI – magnetic resonance imaging
