# Supplementary material for: Understanding parental perspectives on outcomes following paediatric encephalitis: A qualitative study
Source: PLoS One. 2019 Sep 16;14(9):e0220042. doi: 10.1371/journal.pone.0220042 (PMC6746349; doi:10.1371/journal.pone.0220042)
Supplement: S2 Appendix — (DOCX) [file pone.0220042.s002.docx]

**S2 Appendix: Core Outcomes Study topic guide**

1. When they were first diagnosed, what difficulties did they have and what problems were you most concerned about?

• What were they most concerned about?

• Were there any problems that you or they were initially concerned about that improved or became less important during their hospital stay?

• Were there any problems that became more obvious or more important during their hospital stay?

• Any concerns and expectations related to medication?

• At the time of discharge from hospital, what was your priority, what was your child’s priority?

1. Has anything changed for your child as a result of encephalitis, since they were discharged from hospital

• What (if any) problems are currently most important to them?

• Are they having any on-going treatment?

• Is there anything that they need help with now that they didn’t before?

• Have any problems improved or become less important since discharge?

• Have any problems become worse or more important since discharge?

• Have there been any effects on day to day life/relationships and social life?

• Any effect on school?

• Have there been any positive effects of encephalitis for your child?

1. I would now like to discuss any effects that encephalitis may have had on other family members/ family life.

• Have there been any effects on yourself or their mother/father?

• Have there been any effects on other children (if applicable)?

• Have there been any changes to the family’s daily routine?

• Has encephalitis had any impact on the family’s finances (for example: this could be additional costs or time taken off work)?

• Have any of these issues changed over time?

• Have there been any positive effects of encephalitis for your family?

4. How do you feel about your child’s future?

• What are your priorities for your child at the moment? What would you have said if I had asked that question before your child’s illness?

• What do you think your child’s priorities are at the moment? What would you have said if I had asked that question before your child’s illness?

• What do you think are your child’s aspirations for the future? What would you have said if I had asked that question before your child’s illness?

• Has encephalitis affected your expectations for your child’s future?

5. If doctors could offer a new medicine for children with encephalitis, what would you want it to be able to do?

6. Would you say that encephalitis has had a mild/moderate or severe impact on your child and what makes you say that?

Notes

a) Prompts/questions1-4 corresponded to umbrella topics which were included in every interview. The bullet points underneath were used as additional prompts, if necessary, depending on what the parent narrative included.

b) Question 5 was omitted from interview 4 onward as parents were finding it difficult to understand the question and it was not generating an open discussion.

c) Question 6 was added from interview 2 onwards as means of concluding the interview by asking for the parents’ overall assessment of the impact of encephalitis on their child.
